# CISD2 Attenuates Inflammation and Regulates Microglia Polarization in EOC Microglial Cells—As a Potential Therapeutic Target for Neurodegenerative Dementia

**DOI:** 10.3389/fnagi.2020.00260

**Published:** 2020-08-26

**Authors:** Muh-Shi Lin

**Affiliations:** ^1^Division of Neurosurgery, Department of Surgery, Kuang Tien General Hospital, Taichung, Taiwan; ^2^Department of Biotechnology and Animal Science, College of Bioresources, National Ilan University, Yilan, Taiwan; ^3^Department of Biotechnology, College of Medical and Health Care, Hung Kuang University, Taichung, Taiwan; ^4^Department of Health Business Administration, College of Medical and Health Care, Hung Kuang University, Taichung, Taiwan

**Keywords:** CISD2, M1/M2 microglia polarization, anti-inflammatory effects, aging, neurodegenerative disease and dementia

## Abstract

**Background**: Accumulating evidence has demonstrated a significant association between microglia-driven inflammation in the brain and neurodegenerative dementia. We previously showed a significant decline in CISD2 expression in mice models with advanced age. Moreover, we observed that the knockdown of CISD2 led to remarkable inflammation and mitochondrial dysfunction in neural cells. In the present study, we investigated whether CISD2 attenuation influences anti-inflammatory effects and M1-M2 polarization in microglia.

**Materials and Methods**: The knockdown of CISD2 expression by siRNA (siCISD2) in EOC microglial cells was performed to mimic the age-driven decline of CISD2 expression. The extent of the inflammatory reaction, polarization in the M1/M2 spectrum, and NFκB activation were verified in EOC microglial cells exhibiting CISD2 deficiency.

**Results**: In the cellular model of microglia, loss of CISD2 function mediated by siCISD2 exhibited a significant augmentation of proinflammatory signaling, as well as reduced expression levels of Arg-1, Ym1, IL-10, and BCL2. Attenuation of CISD2 expression led to a decrease in the proportion of the M2 phenotype of microglia (compared to M1). Enhanced DNA-binding activity of the NFκB p65 subunit was confirmed in cells transfected with siCISD2, as demonstrated by enzyme-linked immunosorbent assay (ELISA).

**Conclusions**: To the best of our knowledge, this is the first report examining the following phenomena: (1) anti-inflammatory effects of CISD2 in microglia *via* NFκB regulation; and (2) microglial CISD2 assistance in the restoration of M2 microglia phenotype. The anti-inflammatory effects of CISD2 in microglia eventually augment anti-apoptotic effects, which provides a rationale for the development of potential therapeutic target for neurodegenerative diseases and neurodegenerative dementia.

## Introduction

Dementia is a chronic disease leading to disability and dependency among the elderly worldwide. For clinical classification, dementia can be divided into the following major categories: Alzheimer’s disease (AD), vascular dementia (VaD), dementia with Lewy bodies (DLB), frontotemporal dementia (FTD), and mixed dementia (Raz et al., 2016). Due to the complexities of neurodegenerative dementia, precise diagnosis remains a challenge in daily clinical practice. Eventually, patients with dementia will present a catastrophic situation for the entire community and society, leading to highly expensive long-term medical care. Moreover, evidence indicates that sustained brain inflammation due to excessive microglial activation contributes to cerebral aging (Hickman et al., 2018; Sanada et al., 2018), neurodegenerative disease (Maragakis and Rothstein, 2006; Hickman et al., 2018), and neurodegenerative dementia (Meraz-Ríos et al., 2013; Bhaskar et al., 2014).

Microglia stabilize and balance the central nervous system (CNS) by mediating immune responses and providing neurotrophic support for neurons (Wake et al., 2011). Embryologically, microglia are derived from the same origin as peripheral macrophages (myeloid progenitors), whereas other resident CNS cells (e.g., astrocytes, oligodendrocytes, and neurons) are derived from neuroepithelial precursors (Zhang et al., 2018). Microglia can be regarded as a specialized macrophage in the CNS owing to the aforementioned close association. Microglia exert biphasic effects that either benefit the neuron survival (M2 phase) or undermine it (M1 phase) under microenvironmental changes. As such, it is representative of the spectrum of M1 (proinflammation)/M2 (anti-inflammation) phenotypes (Song and Suk, 2017; Zhang et al., 2017). In the microenvironment of the CNS, M2 microglia function to maintain CNS homeostasis and neural protection by moving dynamically throughout the entire CNS parenchyma and performing phagocytosis when they encounter pathological changes initiated by pathogens, plaques, protein aggregates, and infectious agents (Thomas et al., 2015). However, under the circumstances of neurodegeneration (non-stressed status) or CNS trauma (injury-challenged status), temporally inappropriate, prolonged, or excessive inflammation indicates the pathological activation of detrimental M1 microglial cells involving the release of cytokines and chemokines (Tator and Fehlings, 1991; Luo et al., 2002). Aberrantly stimulated microglia produce nitric oxide (NO) in conjunction with reactive oxygen species (ROS), which can amplify the inflammatory cascade. In a deleterious pathological cycle, cytokines promote inflammation by inducing the production of additional cytokines, chemokines, NO, and ROS (Li et al., 2006). Sustained microglia-driven inflammation can eventually lead to apoptosis and the killing of oligodendrocytes and neurons, which can impair neuronal functioning and lead to permanent neurological damage (Wood, 1995; Mattson, 2000; Byrnes et al., 2007). Any novel strategies involving the attenuation of microglial inflammatory response could be beneficial in the management of neurodegenerative disease and dementia.

CISD2 (CDGSH iron-sulfur domain 2) has garnered interest for its protective role against calcium excitotoxicity (Shen et al., 2017), apoptosis (Chen et al., 2009), and inflammation (Lin et al., 2015). *CISD2* has been referred to as a longevity gene, aiding in the preservation of mitochondrial integrity, thereby preventing mitochondrial malfunction and, ultimately, cell death. In response to stress, CISD2 exerts protective effects against autophagy/apoptosis by enhancing BCL2-BECN1 interactions and by the formation of a complex with BCL2 (Chang et al., 2010). Moreover, CISD2 attenuates the excitotoxic Ca^2+^ surge at the endoplasmic reticulum by binding to BCL2 and the inositol 1,4,5-triphosphate receptor (Chang et al., 2012). Furthermore, overexpression of CISD2 has been demonstrated to attenuate neuronal loss and β-amyloid-induced mitochondrial dysfunction in the AD mouse model (Chen et al., 2020). Thus, CISD2 holds promise as a potential therapeutic target for neurodegenerative dementia.

In a previous study, we identified a potential role of CISD2 in exhibiting the anti-inflammatory effects. In a CISD2 knockdown model developed with siCISD2, we observed that CISD2 deficiency led to a significant increase in iNOS production and a reduction in BCL2 levels in SH-SY5Y cells stimulated with lipopolysaccharide (LPS; Lin et al., 2015). No previous study has been reported on the anti-inflammatory effects of CISD2 in microglia. In the current study, we sought to delineate the protective effect of CISD2 in microglial inflammatory reactions associated with neurodegenerative dementia. It is a preliminary investigation into the anti-inflammatory effects of CISD2 in the EOC microglial cell culture model with a specific aim of determining whether it plays a role in the alternation of microglial M1/M2 polarization.

## Materials and Methods

### Cell Lines

Microglia cell lines (EOC 13.31, BCRC 60490) were obtained from the Bioresource Collection and Research Center (BCRC, Hsinchu, Taiwan). EOC 13.31 cell line derived from the brain of a 10-day old female mouse (*Mus musculus*) is dependent on the colony-stimulating factor-1 (CSF-1) for cell proliferation. EOC microglial cells were cultured in 70% Dulbecco’s modified essential medium (DMEM), 10% fetal bovine serum (FBS), and 20% LDMAC conditioned medium derived from LADMAC cells (BCRC 60489, a bone marrow monocytic cell line derived from adult mouse, *M. musculus*). LDMAC conditioned medium containing CSF-1 secreted from LADMAC cells was collected from confluent cultures and filtered with 0.2-mm syringe filters before addition to EOC cell cultures.

### Reverse-Transcription Polymerase Chain Reaction and Real-Time Quantitative Reverse-Transcription Polymerase Chain Reaction

Total RNA was prepared by directly lysing the cultured cells in extraction buffer (Trizol/phenol/chloroform) and reverse transcribing the mRNA into cDNA using oligo-dT and SuperScript II reverse transcriptase (Invitrogen, Carlsbad, CA, USA). The cDNA was subjected to polymerase chain reaction (PCR) for the measurement of mRNA levels of CISD2, BCL2, and M2 microglia specific markers based on a previous report [Pei et al., 2017; IL-10, Arginase-1 (Arg-1), Chitinase-3-like-3 (Ym1)]; proinflammatory mediators including TNF-α, IL-1β, iNOS, and COX2 (representative of M1 microglia); and the housekeeping gene GAPDH (internal control). The PCR protocol involved 25 cycles of denaturation for 1 min at 94°C, annealing for 1 min at 55°C to 60°C, and extension for 1 min at 72°C. The primers used for PCR are listed in Table 1.

**Table 1:** Primers sequences used in the study.

Gene	Orientation	Sequence
CISD2	forward	5′-AAAATCCCAAGGTGGTGAATGA-3′
	reverse	5′-GGACCGCCAGCACCTACA-3′
GAPDH	forward	5′-GGCAAATTCAACGGCACAGT-3′
	reverse	5′-CGCTCCTGGAAGATGGTGAT-3′
TNF-α	forward	5′-GGCACTCCCCCAAAAGATG-3′
	reverse	5′-GCCACAAGCAGGAATGAGA
IL-1β	forward	5′-AAGATGAAGGGCTGCTTCCA-3′
	reverse	5′-ATGTGCTGCTGCGAGATTTG-3′
iNOS	forward	5′-CCTCAGTTCTGCGCCTTTG-3′
	reverse	5′-GTTCGTCCCCTTCTCCTGTTG-3′
COX2	forward	5′-ACAAGCACAATAGACGCACAAGA-3′
	reverse	5′-GGGAGGGCAATTATGATAAGGAT-3′
Arg-1	forward	5′-CGCCTTTCTCAAAAGGACAG-3′
	reverse	5′-CCAGCTCTTCATTGGCTTTC-3′
Ym1	forward	5′-ACCAGTTGGGCTAAGGACAG-3′
	reverse	5′-TGGCCAGGAGAGTTTTTAGC-3′
IL-10	forward	5′-AAGGACCAGCTGGACAACAT-3′
	reverse	5′-TCCTGAGGGTCTTCAGCTTC-3′
BCL2	forward	5′-TGGGATGCCTTTGTGGAACT-3′
	reverse	5′-CAGCCAGGAGAAATCAAACAGA-3′

For real-time quantitative reverse-transcription (qRT)-PCR, the cDNA samples were analyzed with SYBR Green Gene Expression System (ABI PRISM 7300 HT real-time PCR system; Applied Biosystems, Foster City, CA, USA). Minor groove binding dyes and primers for the detection of the genes of interest and GAPDH were designed by ABI. We further determined the threshold cycle (Ct), and the Ct values of proposed genes were normalized using GAPDH as an internal control. The measurements were performed in triplicates.

### Immunoblotting

Total protein was extracted from cultured EOC microglial cells in a lysis buffer containing 20 mM Tris-HCl, 0.1% sodium dodecyl sulfate (SDS), 0.8% NaCl, and 1% Triton-X 100). Electrophoresis with 12% gradient gel was performed to separate the protein extracts. Proteins that underwent electrophoresis were electro-transferred to a nitrocellulose membrane prepared with blocking reagent and primary antibodies [anti-CISD2 (1:500; Thermo Scientific, PA5-34545); anti-iNOS (1:2,000; Thermo Scientific, PA3-030A); anti-Arg-1 (1:5,000; Thermo Scientific, PA5-29645); anti-GAPDH (1:500; Millipore, Billerica, MA, USA)] at 4°C for 12 h before washing and incubation with goat-anti-rabbit IgG HRP (horseradish peroxidase)-conjugated secondary antibodies (Merck Millipore, Cat. #12-348) for 1 h. Chemiluminescence detection was performed following the established protocols (Merck Millipore, WBKLS0500). Bands of interest were visualized and quantified using ImageQuant^TM^ LAS 4000 (GE Healthcare Life Sciences, Marlborough, MA, USA).

### Short Interfering RNA-Mediated RNA Interference

Short interfering RNA (siRNA) specific to CISD2 mRNA was used to attenuate the expression of CISD2 in cultured microglia. This method was similar to that used in our previous research conducted on gene knockdown with minor modifications (Lin et al., 2011a,b, 2015, 2019). EOC microglial cells were transfected using a set of siRNAs specific to CISD2 or scrambled RNA (Silencer^®^ Pre-designed siRNA, Ambion, Austin, TX, USA) using Lipofectamine^TM^ 2000 reagent (Invitrogen, Carlsbad, CA, USA). Briefly, EOC cells were randomly assigned to the experimental groups after being cultured for 5 days. Lipofectamine^TM^ 2000 reagent and siRNA were individually mixed in serum-free medium for 10 min. Lipofectamine transfection reagent was mixed with dissolved siRNA in a serum-free medium for 10 min. Transfected cell mixtures containing 2 × 10^6^ cells were added to each plate.

Five hours after transfection, the medium containing Lipofectamine 2000 was replaced with a microglial culture medium and allowed to stand for an additional 7 h. Real-time qRT-PCR was performed for the detection of CISD2 expression to verify the effectiveness of knockdown.

### Detection of NFκB p65 Activity

NFκB p65 activity was characterized using the NFκB p65 Transcription Factor Assay Kit (ab133112, Abcam, MA, USA) as per the manufacturer’s instructions. Briefly, the extracted nuclear protein was collected for characterizing intracellular p65-NFκB activity by using a spectrophotometer reader to measure absorbance at an optical density (OD) of 450 nm. All measured values were derived with Synergy HT (BioTek, VT, USA).

### Statistical Analysis

The normality test of variables was used to determine whether to use parametric tests for variables with a normal distribution or nonparametric tests for variables with a non-normal distribution. The *p*-values of the normality test for variables of the experimental groups exceeded 0.05. Hence, parametric tests were used for mean comparisons among the experiment groups. Independent two-sample *t*-tests were subsequently used to compare the experiment groups. Statistical analysis was performed using GraphPad Prism software 5.0 (GraphPad Software, Inc., La Jolla, CA, USA).

## Results

### Knockdown of CISD2 Expression Augmented Inflammatory Signaling in EOC Microglial Cells and Enhanced M1 Microglia Polarization

High inflammation due to microglial activation has been demonstrated underlying the pathogenesis of various neurodegenerative diseases in the CNS and neurodegenerative dementia (Thomas et al., 2015; Raz et al., 2016). In our previous study, we demonstrated that the attenuated CISD2 expression is associated with the aging process and leads to the inflammatory response (Lin et al., 2015, 2019). Hence, we sought to determine whether CISD2 deficiency in microglia results in the inflammatory cascade as well as the likelihood that these detrimental insults influence microglia polarization. The CISD2 expression was attenuated in the microglial EOC cell lines *via* siCISD2-mediated knockdown to delineate the inflammatory mechanism involving CISD2 and evaluate the possibility of influencing polarization in the microglia M1/M2 spectrum.

**Figure 1:**
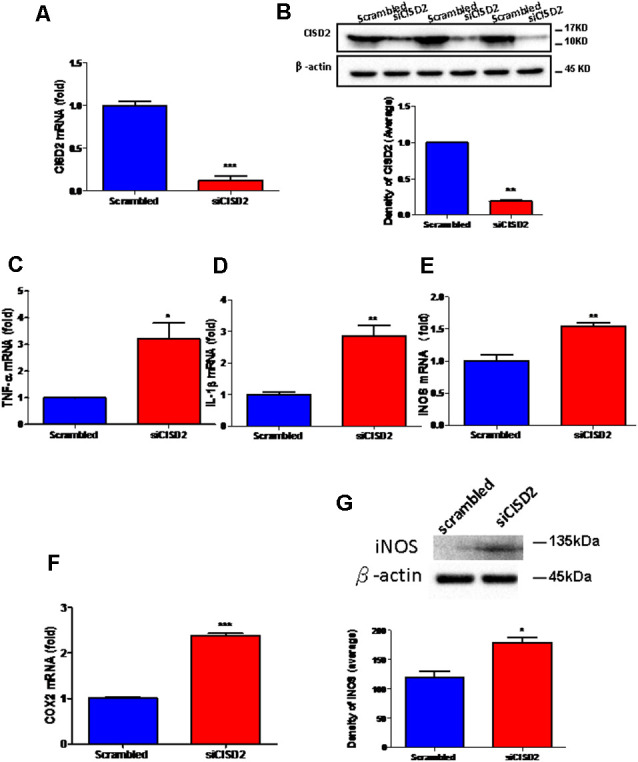
Knockdown of CISD2 expression augmented tendency for M1 microglial polarization and enhanced proinflammatory response in non-stressed EOC microglial cells. The conditions were set as follows: (i) scrambled RNA-transfected control cells; (ii) siCID2-transfected cells. **(A)** Confirmation of knockdown efficiency of CISD2 mRNA and protein **(B)** expression in microglia. **(C)** Expression of TNF-α mRNA, **(D)** IL-1β mRNA, **(E)** iNOS mRNA, and **(F)** COX2 mRNA under all conditions. **(G)** iNOS protein expression in siCID2-transfected EOC microglial cells. The upper panel demonstrates the immunoblot results of iNOS at 135 kDa, and β-actin (45 kDa) serves as an internal control; the lower panel indicates the mean (±SEM) of iNOS/β-actin band intensity in ratio with the control group. Vertical bars indicate the mean ± (SEM) of mRNA or protein expression (*n* = 3). *p < 0.05; **p < 0.01; ***p < 0.001 indicate statistically significant difference when compared to scrambled control cells.

The knockdown efficiency of CISD2 siRNA in the EOC microglial cell line was confirmed by real-time qRT-PCR (*p* < 0.001, labeled as ***, Figure 1A) and western blot analysis (*p* < 0.01, labeled as **, Figure 1B). Each experiment was performed in triplicate.

We observed that mRNA expression levels of proinflammatory mediators were higher in siCISD2-transfected EOC microglial cells than those in scrambled RNA-transfected cells as determined by real-time qRT-PCR (TNF-α, *p* < 0.05, labeled as *, Figure 1C; IL-1β, *p* < 0.01, labeled as **, Figure 1D; iNOS, *p* < 0.01, labeled as **, Figure 1E; and COX2, *p* < 0.001, labeled as ***, Figure 1F). As shown in Figure 1G, western blot analysis confirmed that CISD2 deficiency significantly increased the protein production of iNOS (^*^p < 0.05) in EOC microglial cells.

These results indicate a trend toward M1 microglia-associated inflammatory effects observed with CISD2 attenuation. Thus, CISD2 inactivity could be associated with the proinflammatory effects of M1 microglia underlying the pathological mechanism of neurodegenerative disease and dementia.

### CISD2 Deficiency Reduced the Proportion of M2 Phenotype in EOC Microglial Cells

Under pathological conditions in the CNS, such as aging, and neurodegenerative disease, neuroprotective M2 microglia underwent excessive stimulation, switched to the detrimental M1 phenotype, and subsequently produced various proinflammatory mediators that might lead to irreversible neurological deficits. We observed that CISD2 deficiency in EOC microglial cells led to a broad range of proinflammatory reactions. Next, we sought to determine whether CISD2 attenuation halts protective M2 microglia polarization.

**Figure 2:**
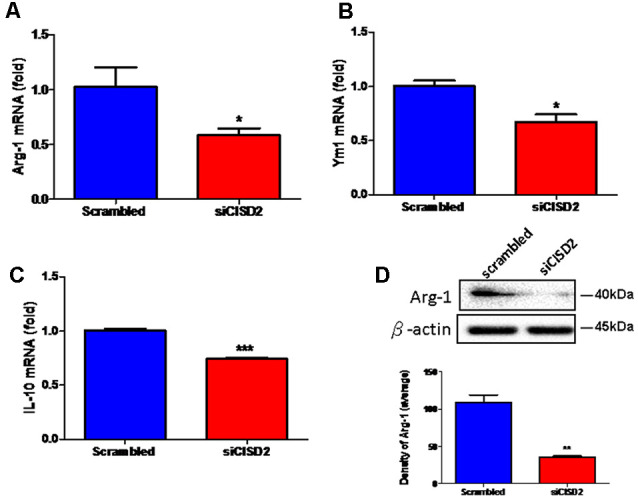
Knockdown of CISD2 expression downregulated **(A)** Arg-1, **(B)** YM1, **(C)** IL-10 mRNA expression, and **(D)** Arg-1 protein expression in non-stimulated EOC microglial cells. In **(A–C)**, data indicate means (±SEM) of fold decrease of Arg-1, YM1, and IL-10 mRNA in comparison to that in the scrambled control group analyzed by real-time qRT-PCR in siCID2-transfected groups (*n* = 3 in each group). *p < 0.05; ***p < 0.001 as compared with scrambled control. In **(D)**, the upper panel demonstrates the immunoblot results of Arg-1 at 40 kDa, and β-actin serves as an internal control; the lower panel indicates the mean (±SEM) of Arg-1/β-actin band intensity in ratio with the scrambled control (*n* = 3). **p < 0.01 indicates the significant difference compared with scrambled control.

Real-time qRT-PCR analysis demonstrated that the mRNA expression of Arg-1, Ym1, and IL-10 was reduced in siCISD2-transfected EOC microglial cells (Arg-1, *p* < 0.05, labeled as *, Figure 2A; Ym1, *p* < 0.05, labeled as *, Figure 2B; and IL-10, *p* < 0.001, labeled as ***, Figure 2C, respectively) compared to the cells subjected to scrambled RNA-transfection. Western blot analysis was used to examine Arg-1 protein expression in EOC microglial cells. Each experiment was performed in triplicates. The siCISD2-transfected cells demonstrated a marked decrease in the protein expression of Arg-1 (*p* < 0.01, labeled as **, Figure 2D) compared to that in the scrambled RNA-transfected cells.

These findings demonstrate that the knockdown of CISD2 tends to reduce the relative proportion of M2 microglia. Thus, it can be concluded that CISD2 deficiency impairs anti-inflammatory effects manifesting as either an enhancement of the detrimental effects associated with M1 or M2-associated protective effects under non-stressed conditions. These results illustrate the protective effects of microglial CISD2 exhibiting anti-inflammatory functions and leading to the enhancement of M2 polarization.

### CISD2 Deficiency Augmented Inflammatory Response *via* NFκB Activation and Promoted Cellular Apoptosis in EOC Microglial Cells

**Figure 3:**
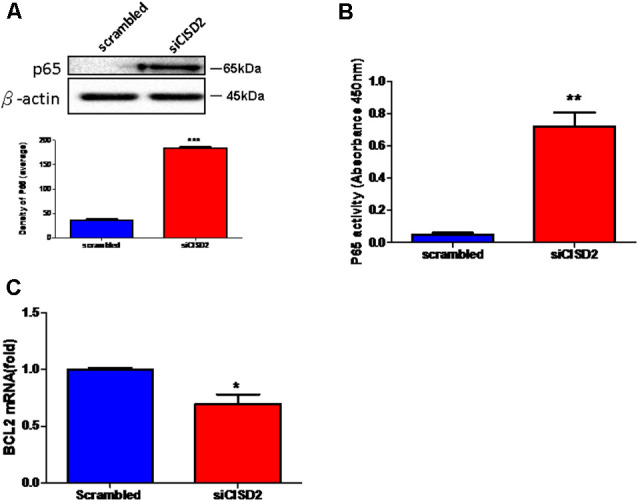
Knockdown of CISD2 expression augmented activation of NFκB and enhanced apoptosis in non-stressed EOC microglial cells.** (A)** Expression levels of p65 (total) were determined by western blot analysis (*n* = 3 in each group). **(B)** Evaluation of P65-NFκB DNA binding activity. The nuclear proteins were prepared, and the NFκB DNA binding activity was analyzed by ELISA (*n* = 3 in each group). **(C)** The extent of anti-apoptosis reflecting BCL2 mRNA expression was determined by real-time qRT-PCR. Vertical bars indicate the mean ± (SEM) of mRNA or protein expression (*n* = 3). *p < 0.05; **p < 0.01; ***p < 0.001 indicate statistically significant difference compared to scrambled control cells.

Finally, we wanted to confirm whether the proinflammatory response induced by CISD2 attenuation involved NFκB signaling in microglial cells. As shown in Figure 3A, western blot analysis demonstrated a significant elevation of the total expression of p65 protein in the siCISD2-transfected group (***p < 0.001), compared to that in the control group that was subjected to scrambled RNA-transfection. Enzyme-linked immunosorbent assay (ELISA) was used to verify the NFκB p65 subunit DNA-binding activity to evaluate the nuclear translocation of NFκB p65. It was observed that P65-NFκB DNA binding activity was significantly higher in siCISD2-transfected EOC microglial cells (**p < 0.01, Figure 3B) than that in the scrambled RNA-transfected cells. Moreover, real-time qRT-PCR analysis demonstrated that the mRNA expression of BCL2 was significantly decreased in siCISD2-transfected EOC microglial cells (*p* < 0.05, labeled as *, Figure 3C), compared to that in scrambled RNA-transfected cells.

In conclusion, these results strongly indicate that microglial CISD2 inhibits the inflammatory response *via* NFκB signaling and possibly by the preservation of M2 polarization in microglia. We postulate that BCL2-associated apoptosis initiated by age-driven CISD2 decline in microglia, which potentially impairs cell survival, can likely be attributed to NFκB activation and neuroinflammation following the downregulation of microglial CISD2.

## Discussion

**Figure 4:**
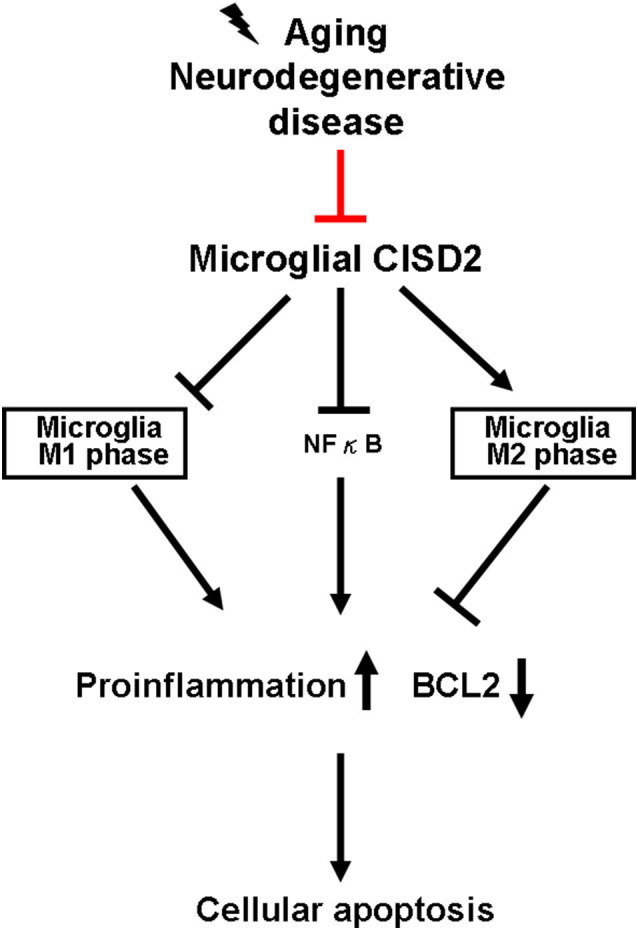
Microglial CISD2 inhibited neuroinflammation and preserved protective microglia M2 phenotype, thereby preventing apoptosis. Microglia-driven chronic inflammation denoted pathological conditions in aging and neurodegenerative disease. Microglial CISD2 can be attenuated during the above-mentioned pathological conditions (as indicated with red bar). The decline in microglial CISD2 expression enhanced and reduced the proportion of detrimental M1 and beneficial M2 phenotype in microglia, respectively. The influence of microglial polarization exacerbated inflammation, elevated the expression of downstream proinflammatory cytokines/chemokines, and consequently augmented apoptosis as indicated by the decrease in BCL2, which potentially led to a detrimental effect on cell survival.

As many protective molecules in nature exhibiting biphasic effects (Lin et al., 2011a,b), microglia-mediated neuroinflammation has been described as a double-edged sword (Hu et al., 2015). It demonstrates protective and restorative effects in the nonpathological and healthy CNS. However, it may also exacerbate and destroy under excessive inflammation under microenvironments of non-stressed (as a proxy for chronic inflammation driven by aging and neurodegenerative disease) and injury-challenged CNS (as a proxy for high-grade impact from neurotrauma). During the early stages of neural pathological conditions of the CNS (e.g., aging and neurodegenerative disease), protective M2 microglia tend to be dominant in the spectrum of proinflammatory/anti-inflammatory phenotypes. During low-grade inflammation, the activated M2 microglia scavenge fatal pathogens to confer neuronal protection. However, when the damage exceeds the range of homeostasis, a decline is observed in the proportion of activated M2 microglia. Accordingly, a delayed reactivation of detrimental M1 microglia can lead to a deleterious inflammatory cycle (Karve et al., 2016), which can be detrimental to neuronal survival and potentially cause CNS disease, such as dementia. Figure 4 presents a schematic illustration showing the proposed sequence of events associated with the anti-inflammatory effects of CISD2 in microglia, as determined in this study.

During the process of aging, the cerebral immune system remains relatively active resulting in the upregulation of inflammatory genes associated with the aging brain. Under such an immunocompetent response, cerebral microglia are primed to be activated and remain resistant to immune adaptive regulation (Norden and Godbout, 2013). This type of sustained microglial dysfunction can cause low-grade chronic neuroinflammation in the brain and thereby exacerbate the cerebral aging process (Simen et al., 2011). Unlike normal aging, neurodegenerative diseases (due to genetic or environmental influences) can lead to misfolding and accumulation of native cerebral proteins, such as prion, tau, β-amyloid, α-synuclein, and huntingtin. Conformational changes in these proteins are resistant to proteolysis resulting in the formation of so-called inclusion bodies in the brain (Jucker and Walker, 2011). When stimulated by toll-like receptors, the aggregation of such atypical proteins promotes the release of microglial-derived inflammatory mediators at high concentrations resulting in long-lasting inflammatory stimuli. Such effects are observed in AD, multiple sclerosis (MS), and Parkinson’s disease (PD; Ross and Poirier, 2004). Thus, microglia-driven inflammatory cascades have definitively been linked to aging, neurodegenerative diseases, and neurodegenerative dementia.

The precise function of CISD2 has not been elucidated. Interestingly, CISD2 expression is attenuated during non-stressed or injury-challenged conditions *in vivo* and *in vitro* (Lin et al., 2019). We observed a significant reduction in CISD2 expression with age in the mouse brain and spinal cord (Lin et al., 2019). In mouse models, spinal cord injury has been demonstrated to attenuate the expression of CISD2 (Lin et al., 2015). Moreover, we previously observed that CISD2 deficiency exacerbated inflammatory responses and mitochondrial dysfunction in cultured cells (with or without injury). Specifically, CISD2 expression was lower in non-stressed 35 *DIV* astrocytes than in 7 *DIV* cells, and the expression of iNOS along with RANTES (regulated on activation, normal T cell expressed and secreted) was higher (Lin et al., 2019). In LPS-stimulated CISD2 knockdown SH-SY5Y cells, the deficiency of CISD2 led to elevated iNOS levels and significant mitochondrial dysfunction (Lin et al., 2015, 2019). Resident immune cells (i.e., microglia) would be the apparent focus while investigating inflammatory cascades in the CNS.

Priming and activation in microglia can promote the proinflammatory signaling associated with aging, neurodegenerative disease, and traumatic CNS injury (Norden et al., 2015). Data obtained in this study demonstrated that CISD2 deficiency (induced by the knockdown of CISD2 expression) augmented the detrimental effects associated with the M1 phase and led to neuroinflammation and apoptosis in non-stimulated EOC microglial cells. Hence, we postulate that CISD2 exerts anti-inflammatory effects in microglia under non-stressed microenvironments (as a proxy for microglial inflammation driven by aging and neurodegenerative dementia). The attenuation of microglia-mediated inflammatory cascades can be beneficial under such pathological conditions. The beneficial effect of the dementia-CISD2-microglial anti-inflammation axis provides a strong rationale for further development of CISD2-based anti-inflammatory therapies for patients with the aforementioned disorders.

Our data provide novel evidence that the anti-inflammatory effect of microglial CISD2 is associated with the regulation of NFκB signaling. At present, the molecular mechanism underlying the anti-inflammatory effects of CISD2 has yet to be elucidated. When located in the cytoplasm and mitochondrial intermembrane space, the activation of NFκB, in addition to a role as well-recognized proinflammatory mediator, has been linked to inflammation, mitochondrial dysfunction, and eventual cellular apoptosis (Albensi, 2019). Thus, it can be interpreted that the attenuation of CISD2 owing to primary insults from neurotrauma or inflammation from CNS disease inhibits the protective effects against NFκB in cytoplasm and mitochondria leading to a remarkable inflammatory response and mitochondrial dysfunction. Furthermore, evidence has been obtained stating that calcium regulates the expression of TNF-α (Canellada et al., 2006) and modulates macrophage-driven inflammatory cascades (Racioppi et al., 2012). We posit that anti-inflammatory effects may be attributed to the regulatory role of CISD2 in calcium metabolism (Chang et al., 2012).

It has been suggested that CISD2 combines with BCL2 to antagonize the apoptotic mediator Beclin 1 (Chang et al., 2012). NFκB also regulates apoptotic effects *via* the combination or dissociation of Beclin 1/Bcl2 complex (Salminen et al., 2012). Moreover, elevated activity of NFκB transcription factors has also been demonstrated to modulate the expression of BCL2 (Chen et al., 2015). In this study, CISD2 was demonstrated to mediate the upstream regulation of NFκB, as observed by the DNA-binding activity of the NFκB p65 subunit. Collectively, the results indicate that CISD2 plays an upstream regulatory role in the functions of NFκB and BCL2. Advanced inflammation and mitochondrial dysfunction are highly likely to result in cell damage and negative cell survival. This may explain why CISD2 deficiency leads to potent inflammatory and apoptotic signaling.

As located in the outer membrane of mitochondria, CISD2 deficiency has been demonstrated to cause mitochondrial dysfunction and may undermine mitochondrial integrity. Note that mitochondrial dysfunction has been shown to inhibit M2 microglial polarization (Nakagawa and Chiba, 2014). It has been reported that M1 macrophages undergo aerobic glycolysis and pentose phosphate pathway (PPP) in obtaining a supply of energy. Conversely, M2 macrophages use acetyl-CoA and participate in the mitochondrial TCA (tricarboxylic acid) cycle (Culmsee et al., 2019). Thus, CISD2 deficiency-driven mitochondrial dysfunction contributes to a decrease in TCA cycle activities and augments aerobic glycolysis and the PPP pathway. In so doing, it leads to the promotion of the M1 phenotype along with the reduction of the M2 phenotype.

Some limitations have been attributed to this study. First, based on some previous novel findings involving non-immune cells (SH-SY5Y), we used the cell culture model developed with EOC microglial cell line to evaluate the mechanisms in this study. In future studies, it would be prudent to examine this putative mechanism in primary cultured microglia or *via in vivo* research using animal or human tissue. Moreover, the mechanism underlying the effect of CISD2 knockdown on M1/M2 microglia polarization will have to be verified using morphological analysis conducted *in vitro* or *in vivo*, such as staining for microglia M1/M2 profile marker. We demonstrated the involvement of CISD2 in the regulation of inflammatory genes under basal conditions in the present study using siCISD2 to attenuate CISD2 expression. Confirmation of this mechanism will be considered in further studies demonstrating the overexpression of CISD2 in microglia.

## Conclusion

The results obtained in this study demonstrate that CISD2 exerts anti-inflammatory effects, which may be induced by the suppression of NFκB activation and the preservation of the protective M2 phenotype in non-stressed microglia. CISD2 deficiency in microglia could be one of the mechanisms underlying cell apoptosis, perhaps occurring *via* inflammation. CISD2-based anti-inflammatory therapy is a strong candidate for the treatment of neuroinflammation-driven aging and neurodegenerative diseases.

## Data Availability Statement

The raw data supporting the conclusions of this article will be made available by the authors, without undue reservation.

## Ethics Statement

The study was reviewed and approved by the Animal Care and Ethics Committee of National Ilan University.

## Author Contributions

M-SL: writing—original draft, formal analysis, resources, methodology, funding acquisition, writing—review and editing, and conceptualization.

## Conflict of Interest

The author declares that the research was conducted in the absence of any commercial or financial relationships that could be construed as a potential conflict of interest.
